# Reproductive Desire in Women Living with HIV/AIDS with Serodiscordant Partners

**DOI:** 10.3390/ijerph192113763

**Published:** 2022-10-22

**Authors:** Marise Ramos de Souza, Rafael Alves Guimarães, Waldemar Naves do Amaral, Vanessa Elias da Cunha, Brenner Dolis Marretto de Moura, Maria Alves Barbosa, Sandra Maria Brunini

**Affiliations:** 1 Curso de Enfermagem, Universidade Federal de Jataí, Jataí 75801-615, GO, Brazil; 2 Faculdade de Medicina, Universidade Federal de Goiás, Goiânia 74690-900, GO, Brazil; 3 Faculdade de Enfermagem, Universidade Federal de Goiás, Goiânia 74690-900, GO, Brazil; 4 Instituto de Patologia Tropical e Saúde Pública, Universidade Federal de Goiás, Goiânia 74690-900, GO, Brazil

**Keywords:** human immunodeficiency virus, acquired human immunodeficiency syndrome, reproductive desire, Brazil

## Abstract

Objective: To estimate the prevalence and factors associated with reproductive desire in a sample of women living with HIV/AIDS (WLHA) with serodiscordant partners. Study design: Between September 2015 and August 2016, a cross-sectional study was conducted among 110 WLHA from HIV/AIDS treatment services and non-governmental organizations. An interview was conducted using a structured questionnaire to collect sociodemographic data, reproductive desire, and potential predictor variables. Poisson regression was used to analyze the factors associated with reproductive desire in the sample. Results: The prevalence of reproductive desire was 32.7% (95% Confidence Interval: % CI: 24.7–42.0%). In regression analysis, we observed an association between reproductive desire and the following variables: age < 30 years; relationship time < 2 years; reproductive desire for the partner; and absence of children. Conclusions: The prevalence of reproductive desire in the investigated sample was relatively high. Young age and reproductive desire for the partner were the main associated factors.

## 1. Introduction

It is estimated that 36.7 million people are living with HIV/AIDS (PLWHA) worldwide and that 2.1 million new infections occurred in 2015 [1]. Of this population, approximately 80% are of reproductive age (between 15 and 49 years), and, of these, almost half have HIV-negative partners, characterizing serodiscordant relationships [1,2].

Sexual and reproductive health corresponds to social and psychological needs that can help PLWHA improve their health status. Due to access to prevention of vertical transmission and antiretroviral therapy (ART), these individuals are more likely to consider motherhood [3]. The advent of this therapy has led to an increase in the quality of life and life expectancy of PLWHA with what is now considered a non-lethal disease in properly treated persons, reducing the chance of sexual transmission to the partner [4,5]. ART has significantly reduced the vertical transmission of HIV, promoting an increase in reproductive desire in PLWHA, especially women living with HIV/AIDS (WLHA) [6].

The reproductive desire expressed by PLWHA in serodiscordant relationships has long been treated inadequately by health services [7]. For a long time, PLWHA in serodiscordant relationships suppressed their reproductive desire for fear of transmission or lack of knowledge regarding options for motherhood (e.g., assisted reproductive techniques). Some evidence suggests that women whose partners were seropositive had a lower prevalence of reproductive desire than those with seronegative partners [8,9].

A meta-analysis showed that men living with HIV/AIDS had a higher prevalence of reproductive desire than WLHA, although studies that analyze the prevalence and factors associated with stratified reproductive desire for this group of women are scarce [10]. The analysis of reproductive desire in WLHA is fundamental for planning reproductive care for PLWHA, since the decision to have children, in many societies, often stems from maternal desire. Previous studies have shown variations in the prevalence of reproductive desire in WLHA. A study in Ethiopia, Africa, showed a prevalence of 40.3% [11]; in Rwanda, Africa, a prevalence of 40.7% was found [12]; in the United States of America, a desire prevalence of 43.0% was found [13]; in Spain, there was a prevalence of 49.0% [14]. In Brazil, a study in Ceará, northeastern Brazil, showed that 64.0% of MLHA wanted to have children [8]; meanwhile, a study in São Paulo, Southeast region, showed a prevalence of 19.2% in this population [15]. However, most previous studies were conducted in African countries [10] and did not analyze associated factors in women who have serodiscordant partners.

Previously conducted studies have shown multiple determinants of reproductive desire in WLHA, such as young age, absence of children, ART use, CD4 ≥ 350 cells, undetectable viral load, and the partner’s reproductive desire, among others [16,17,18,19].

In Brazil, studies on the determinants of reproductive desire in WLHA are scarce, especially in those with serodiscordant partners [8,20]. The identification of factors associated with reproductive desire is fundamental for interventions aimed at adequately attending to WLHA in serodiscordant relationships, thus reducing the risk of vertical HIV transmission [21]. The objective of this study was to estimate the prevalence and factors associated with reproductive desire in a sample of WLHA with serodiscordant partners.

## 2. Material and Methods

Between September 2015 and August 2016, a quantitative, observational, and cross-sectional study was conducted with WLHA from referral services for HIV treatment and nongovernmental organizations (NGO) in the state of Goiás, in the Center-West region of Brazil. The following inclusion criteria were considered: women with a diagnosis of HIV/AIDS verified in the medical chart and a reproductive age between 18 and 49 years with a serodiscordant partner according to self-report. The exclusion criteria were menopausal status according to women’s self-report, even for those within the study inclusion age range. We excluded persons over 50 years old, considering the definition of reproductive age for women defined by the World Health Organization (18–49 years) [22].

The sampling performed in this study was non-probabilistic for convenience. All women attending the study site were invited to participate (*n* = 310) and after including only WLHA with discordant partners, 110 were included in the study.

Initially, all women were invited to participate in the study while awaiting medical appointments or during NGO meetings. After obtaining written consent, interviews were conducted face to face using a structured questionnaire on sociodemographic data, reproductive desire, and potential associated factors. The questionnaire was applied by previously trained health professionals and tested in a previous pilot study. The questionnaire is available in full in the article by Ramos et al. [18].

### 2.1. Variables

The dependent variable investigated was reproductive desire expressed by the participants at the time of the interview. This variable has been used in previous studies conducted among WLHA [8].

The independent variables analyzed were: age (years), categorized as ≥40, 30–39, and <30 years; education (years), categorized as ≤4, 5–8, and >8 years; formal employment (no or yes) [23]; self-declared race/ethnicity, categorized as white, black, brown, or other (Asian or native Brazilian) according to the race/skin color classification of the Brazilian Institute of Geography and Statistics [24]; alcohol use (current), defined as consumption in the previous month (no or yes); tobacco use (current), defined in the previous month (no or yes); illicit drug use (ever) in lifetime (no or yes); condom use in the previous 12 months (never, sometimes, or always); relationship time, categorized as >5, 3–5, and ≤2 years; undetectable viral load (no or yes), defined as a result less than or equal to 50 copies/mL as available in medical records—those individuals on ART with an undetectable viral load for at least 6 months cannot transmit HIV through sexual intercourse (Undetectable = Untransmittable) [25,26]; most recent CD4 cell count, categorized as <200, 200–350, and ≥350 cells [27]; ART use (no or yes); time since diagnosis with HIV infection, categorized as <2, 3–5, and ≥5 years; opportunistic illness in the last 6 months (defined as those infections that occur as a result of HIV immunosuppression, such as esophageal candidiasis, pneumonia, cytomegalovirus disease, among others (no or yes) [28]; STIs in the previous 6 months (no or yes); history of children with positive HIV serology (no or yes) [27]; number of children, categorized as none, 1–2, and ≥3; having become pregnant after HIV diagnosis (no or yes) [29]; history of abortion (no or yes) [27]; reproductive desire of the partner (no, do not know, or yes); no knowledge of assisted reproduction techniques (no or yes); and knowledge of how to reduce risk of HIV transmission (no or yes). All variables, except CD4 cell count and viral load, were measured using self-report.

### 2.2. Statistical Analyses

The data were analyzed in STATA, version 14.0 (StataCorp LLC, College Station, TX, USA). The normality of the quantitative variables was verified by the Kolmogorov–Smirnov test. Descriptive analysis of variables was performed. Quantitative variables were presented as mean and standard deviation (SD) and qualitative variables as absolute and relative frequencies. Prevalence of reproductive desire was estimated with a 95% confidence interval (95% CI). Bivariate Poisson regression analysis was conducted to verify the factors associated with the dependent variable investigated. Variables with *p* < 0.20 were then included in the Poisson regression model with robust variance to estimate the adjusted prevalence ratio (APR) and respective 95% CI. Variables with *p* values < 0.05 were considered statistically significant.

### 2.3. Ethical Aspects

This study was approved by the Research Ethics Committee of the Federal University of Goiás, protocol number 763.839/2014. Written consent was obtained for all participants.

## 3. Results

A total of 310 WLHA were invited to participate in the study. Of these, 36 were excluded because they were 50 years of age or over, and 69 had no partners. Of those remaining (*n* = 205), 27 did not know the serological status of their partners, and 68 had partners with positive serology for HIV. No woman was excluded due to menopause. Thus, the sample of this study was composed of 110 WLHA with serodiscordant partners.

Table 1 presents the description of the variables investigated in the study. The averages of age and schooling were 40.0 years (SD: 7.8) and 8.3 years (SD: 4.4), respectively. More than half of the participants (55.5%) had formal employment. The use of tobacco, alcohol, and illicit drugs was reported by 17.3%, 40.9%, and 10.9% of women, respectively.

Regarding the clinical characteristics, opportunistic disease and STIs in the previous 6 months were reported by 21.8% and 15.7% of the participants, respectively. Almost all the women (91.8%) were on ART, 75.6% had the most recent CD4 cell count > 350 cells, and 48.9% had undetectable viral load (Table 1).

The prevalence of reproductive desire was 32.7% (95% CI: 24.7–42.0%). In a bivariate analysis, it was verified that the prevalence was 5.41 times higher (PR: 5.41; 95% CI: 2.20–13.27) in women aged < 30 years compared to women aged ≥ 40 years. Being of black race/ethnicity increased the prevalence by 4.10 times (PR: 4.10; 95% CI: 1.18–14.28) when compared to white participants. There was also an association between the dependent variable and the partner’s reproductive desire (PR: 16.11; 95% CI: 3.84–67.51) and absence of children (PR: 4.62; 95% CI: 1.99–10.71) (Table 2).

Table 3 presents the factors associated with reproductive desire after adjustment in the Poisson regression model with robust variance. An association was observed between the reproductive desire and the following variables: age < 30 years (APR: 4.30; 95% CI: 1.92–9.61); relationship time < 2 years (APR: 2.25; 95% CI: 1.01–4.99); reproductive desire in the partner (APR: 8.73; 95% CI: 2.59–29.41); and absence of children (APR: 2.81; 95% CI: 1.10–7.17).

## 4. Discussion

To our knowledge, this is the first study to analyze reproductive desire in a sample of WLHA with serodiscordant partners from the Center-West region of Brazil. The investigation of the reproductive desire of this population presents great implications for partners and babies due to the potential risk of sexual and vertical transmission of HIV [30]. The results showed a prevalence of reproductive desire in WLHA of 32.7%. In addition, we identified that age < 30 years, reproductive desire in the partner, time of relationship < 2 years, and absence of children were determinants of reproductive desire. The wide availability of ART in the Brazilian public system and improvement in quality of life may be associated with the high prevalence of reproductive desire in WLHA with serodiscordant partners found in this research [8].

The frequency of reproductive desire estimated in this investigation was lower than that found in 62 WLHA with serodiscordant partners from Ceará (northeastern region) (49.1%) [8]. It should be emphasized that the methodological differences and other potential sociodemographic characteristics of each sample can explain this result. International studies have shown mixed results in the prevalence of reproductive desire in WLHA with serodiscordant partners. In Uganda, a frequency of 26% was found in this population (*n* = 100) [30]. In the United States of America, a prevalence of 44% was estimated in WLHA with serodiscordant partners (*n* = 102) [16].

In this investigation, young age (<30 years) was strongly associated with reproductive desire. This demographic factor was identified as a determinant of reproductive desire in developed and developing countries regardless of the serological status of the partner [8,31,32]. In addition, the absence of children was a strong predictor of reproductive desire in the investigated sample in findings observed in other studies conducted among WLHA [8,17]. WLHA with serodiscordant partners who are younger and childless, as well as the general population, are at peak reproductive age and continue to want more children until they reach the desired levels of fertility [33].

In the present study, the partner’s reproductive desire was independently associated with the reproductive desire by the WLHA, as found in previous investigations [33,34]. In fact, men play a key role in the reproductive desire of the WLHA [1,34]. For example, in Uganda (Africa), a study conducted among 114 serodiscordant couples showed that the partner’s desire for children was the main determinant of reproductive desire (odds ratio: 24.0) [35]. In the northeastern region of Brazil, a study conducted among WLHA showed a strong association between reproductive desire in the partner and desire for children (odds ratio: 3.35) [8], similar to a result found in other research conducted in the southeast of the country [9]. Qualitative research indicates that the emotional and financial support of partners increases individuals’ reproductive desire [36]. A study conducted in India identified family support as one of the main factors distinguishing women with reproductive desire from those without [37]. Another pilot study conducted among 49 women in South Florida showed that the partner significantly influences the decision of the WLHA to conceive [25]. These results suggest that counseling and design programs should include partners in guidelines on the reproductive health of WLHA [34].

Other factors, such as use of ART, undetectable viral load, and CD4 cell count > 350 cells, were not factors associated with reproductive desire, unlike in other investigations [27]. The small sample number may not have had the statistical power to detect these associations [17]. In addition, almost the entire sample was in ART. Some evidence suggests that the presence of favorable laboratory biomarkers (e.g., CD4+ cell counts or undetectable viral load) and ART use increase the prevalence of reproductive desire in WLHA [17,38]. This is due to the perception of reduced transmission of HIV to the child, improved quality of life, increased life expectancy, and improved perceived and actual health status among PLWHA [35,39]. Moreover, the use of ART helps to restore the immune system and maintain viral load at undetectable levels, thus contributing to an increase in quality of life [40].^.^

An undetectable viral load can influence increased physical health and adherence/access to ART, which increases the feeling of safety of WLHA for pregnancy and increases the prevalence of reproductive desire. The lack of association between undetectable viral load and reproductive desire found in this study, contrary to evidence from other studies [18,19]. These results suggest the need for strengthening the reproductive counseling actions for MLHA with undetectable viral load on reducing the risks of vertical transmission of HIV in cases of viral loads being undetectable. In fact, in this study, 54.6% of women said that they were not aware of reducing the risk of HIV transmission, even though 91.8% of the sample were on ART treatment and 48.9% had an undetectable viral load; thus, suggesting the need for reproductive counseling regarding mother-to-child transmission of HIV, adherence to treatment, and a significant reduction in the risk of transmission in the event of an undetectable viral load.

The present study has some limitations. First, the cross-sectional, rather than longitudinal, nature of the research limits the establishment of cause and effect between reproductive desire and predictor variables. Behavioral (e.g., condom use, psychoactive substances) and some clinical data (e.g., HIV diagnostic time) were self-reported and susceptible to memory and response biases. The small sample size is another potential limitation, which may have contributed to the absence of statistical detection of other determinants of reproductive desire. However, the number of participants in our sample was consistent with previously conducted studies [14,16]. The non-probabilistic sample limited to women in treatment or linked to NGOs limits the generalization of results to other scenarios.

## 5. Conclusions

The results of the study suggest that health professionals who aid WLHA with serodiscordant partners should discuss and systematically address sexual and reproductive health. These actions can help WLHA with serodiscordant partners make decisions about motherhood [11]. Additionally, it is necessary to strengthen family planning services for WLHA, addressing factors associated with reproductive desire and prevention of unwanted pregnancies [38]. In addition, the partner should be involved in counseling since they seem to be the main influencer in the reproductive desire of this population. Finally, studies in other geographical locations that address determinants of reproductive desire in WLHA with serodiscordant partners are necessary to verify the real dimension of reproductive desire in Brazil.

In conclusion, the prevalence of reproductive desire in the sample investigated was relatively high. Young age, reproductive desire of a partner, absence of children, and time of relationship < 2 years were the factors affecting reproductive desire. This study adds data on the reproductive aspects and associated factors with reproductive desire in WLHA with serodiscordant partners in Brazil. The identification of these factors is fundamental for interventions aimed at reproductive counseling in serodiscordant relationships.

## Figures and Tables

**Table 1:** Characteristics of women living with HIV/AIDS with serodiscordant partners, Goiás State, 2015–2016.

Variables	*n* = 110	%
Age (years), mean (SD)	37.0 (7.8)	
≥40	48	43.6
30–39	43	39.1
<30	19	17.3
Education (years), mean (SD)	8.3 (4.4)	
≥4	24	21.8
5–8	38	34.5
>8	48	43.6
Formal employment		
No	49	44.5
Yes	61	55.5
Race/ethnicity		
White	22	20.0
Black	25	22.7
Mixed Brazilian	53	48.2
Others (Asian or native Brazilian)	10	9.1
Tobacco use ^1^		
Yes	19	17.3
No	91	82.7
Alcohol use ^1^		
Yes	45	40.9
No	65	59.1
Illicit drug use ^2^		
Yes	12	10.9
No	98	89.1
Condom use ^3^		
Never	10	9.1
Sometimes	21	19.1
Always	79	71.8
Relationship time (years)		
>5	38	34.5
3–5	31	28.2
≤2	41	37.3
Reproductive desire in partner		
No	50	45.5
Do not know	15	13.6
Yes	45	40.9
Number of children, mean (SD)	2.5 (1.7)	
≥3	54	49.1
1–2	42	38.2
None	14	12.7
Children with positive serology for HIV ^4^		
Yes	2	2.0
No	99	98.0
Pregnancy after diagnosis ^5^		
Yes	39	38.2
No	63	61.8
History of abortion ^5^		
No	71	69.6
Yes	31	30.4
Opportunistic illness ^3^		
Yes	24	21.8
No	86	78.2
STIs ^3,6^		
Yes	17	15.7
No	91	84.3
Time since diagnosis (years)		
≥2	22	20.0
3–5	27	24.5
>5	61	55.5
ART use		
No	9	8.2
Yes	101	91.8
CD4 ^7^		
<200	8	9.8
200–350	12	14.6
>350	62	75.6
Undetectable viral load ^3,8^		
No	45	51.1
Yes	43	48.9
Knowledge of assisted reproduction		
No	68	61.8
Yes	42	38.2
Knowledge of how to reduce risk of HIV transmission		
No	62	56.4
Yes	48	43.6

ART = Antiretroviral therapy; HIV = Human immunodeficiency virus; SD = Standard deviation; STIs = Sexually transmitted infections; ^1^ Current (in the previous month); ^2^ Ever (lifetime); ^3^ Previous 6 months; ^4^ Missing data = 9; ^5^ Missing data = 8; ^6^ Missing data = 2; ^7^ Missing data = 28; ^8^ Missing data = 26.

**Table 2:** Potential factors associated with reproductive desire in women living with HIV/AIDS with serodiscordant partners, Goiás State, 2015–2016.

Variables	Reproductive Desire		Crude PR (95% CI)	*p ^2^*
	*n*/Total ^1^	%		
Age (years)				
≥40	7/48	14.6	1.00	
30–39	14/43	32.6	2.23 (0.90–5.53)	0.083
<30	15/19	78.9	5.41 (2.20–13.27)	<0.001
Education (years)				
≥4	7/24	29.2	1.00	
5–8	10/38	26.3	0.90 (0.34–2.37)	0.835
>8	19/48	39.6	1.35 (0.57–3.22)	0.490
Formal employment				
No	15/49	30.6	1.00	
Yes	21/61	34.4	1.12 (0.57–2.18)	0.728
Race/ethnicity				
White	3/22	13.6	1.00	
Black	14/25	56.0	4.10 (1.18–14.28)	0.026
Mixed-race	18/53	34.0	2.49 (0.73–8.45)	0.143
Others (Asian or native Brazilian)	1/10	10.0	0.73 (0.07–7.04)	0.788
Tobacco use ^3^				
Yes	7/19	36.8	1.00	
No	29/91	31.9	0.86 (0.37–1.97)	0.731
Alcohol use ^3^				
Yes	15/45	33.3	1.00	
No	21/65	32.3	0.96 (0.49–1.88)	0.916
Illicit drug use ^4^				
Yes	7/12	58.3	1.00	
No	2998	29.6	0.50 (0.22–1.15)	0.107
Condom use ^5^				
Never	5/10	50.0	1.00	
Sometimes	6/21	28.6	0.57 (0.17–1.87)	0.335
Always	25/79	31.6	0.63 (0.24–1.65)	0.350
Relationship time (years)				
>5	6/38	15.8	1.00	
3–5	9/31	29.0	1.83 (0.65–5.16)	0.248
≤2	21/41	51.2	3.24 (1.30–8.03)	0.011
Reproductive desire in partner				
No	2/50	4.0	1.00	
Do not know	5/15	33.3	8.33 (1.61–42.95)	0.011
Yes	29/45	64.4	16.11 (3.84–67.51)	<0.001
Number of children				
≥3	10/54	18.5	1.00	
1–2	14/42	33.3	1.80 (0.79–4.05)	0.156
None	12/14	85.7	4.62 (1.99–10.71)	<0.001
Children with positive serology for HIV				
Yes	-/2	-	1.00	
No	28/99	28.3	1.12 (0.27–4.62)	0.868
Pregnancy after diagnosis				
Yes	9/39	23.1	1.00	
No	20/63	31.7	1.37 (0.62–3.02)	0.427
History of abortion				
No	18/71	25.4	1.00	
Yes	11/31	35.5	1.39 (0.66–2.96)	0.380
Opportunistic illness history ^5^				
Yes	7/34	29.2	1.00	
No	29/86	33.7	1.15 (0.50–2.63)	0.730
STIs history ^5^				
Yes	6/17	35.3	1.00	
No	30/91	33.0	0.93 (0.38–2.24)	0.879
Time since diagnosis (years)				
≥2	8/22	36.4	1.00	
3–5 >5	10/27	37.0	1.01 (0.40–2.58)	0.969
>5	18/61	29.5	0.81 (0.35–1.86)	0.623
ART use				
No	3/9	33.3	1.00	
Yes	33/101	32.7	0.98 (0.30–3.19)	0.974
CD4				
<200	3/8	37.5	1.00	
200–350	1/12	8.3	0.22 (0.02–2.13)	0.193
>350	23/62	37.1	0.98 (0.39–3.29)	0.986
Undetectable viral load ^5^				
No	13/45	28.9	1.00	
Yes	16/43	37.2	1.28 (0.61–2.67)	0.498
Knowledge of assisted reproduction				
No	21/68	30.9	1.00	
Yes	15/42	35.7	1.15 (0.59–2.24)	0.667
Knowledge of how to reduce risk of HIV transmission				
No	16/62	25.8	1.00	
Yes	20/48	41.7	1.61 (0.83–3.11)	0.153

95% CI = 95% Confidence interval; ART = Antiretroviral therapy; HIV = Human immunodeficiency virus; PR = Prevalence ratio; SD = Standard deviation; STIs = Sexually transmitted infections; ^1^ Valid answers; ^2^ Wald chi-square test; ^3^ Current (in the previous month); ^4^ Ever (lifetime); ^5^ Previous 6 months.

**Table 3:** Factors associated with reproductive desire in women living with HIV/AIDS with serodiscordant partners, Goiás State, 2015–2016.

Variables	Adjusted ^1^ PR (95% CI)	*p* ^2^
Age (years)		
≥40	1.00	
30–39	2.09 (0.92–4.74)	0.075
<30	4.30 (1.92–9.61)	<0.001
Race/ethnicity		
White	1.00	
Black	1.52 (0.33–7.06)	0.588
Mixed-race	1.33 (0.31–5.62)	0.697
Others (Asian or native Brazilian)	0.79 (0.06–9.25)	0.854
Time of relationship (years)		
>5	1.00	
3–5	1.48 (0.61–3.59)	0.382
≤2	2.25 (1.01–4.99)	0.045
Illicit drug use ^3^		
Yes	1.00	
No	0.79 (0.40–1.58)	0.522
Reproductive desire in partner		
No	1.00	
Do not know	2.69 (0.80–9.05)	0.108
Yes	8.73 (2.59–29.41)	<0.001
Number of children		
≥3	1.00	
1–2	1.01 (0.56–1.84)	0.951
None	2.81 (1.10–7.17)	0.030
CD4		
<200	1.00	
200–350	0.52 (0.09–2.76)	0.446
>350	0.75 (0.29–1.92)	0.559
Knowledge of how to reduce risk of HIV transmission		
No	1.00	
Yes	1.42 (0.72–2.81)	0.305

95% CI = 95% Confidence interval; ART = Antiretroviral therapy; HIV = Human immunodeficiency virus; PR = Prevalence ratio; SD = Standard deviation; STIs = Sexually transmitted infections; ^1^ Model adjusted by age, race/ethnicity, time of relationship, illicit drug use, reproductive desire in partner, number of children, most recent CD4, and knowledge of how to reduce risk of HIV transmission (variables with *p*-value < 0.20 in the bivariate analysis); ^2^ Wald chi-square test; ^3^ Current (in the previous month); R^2^: 0.315.

## Data Availability

The data will be made available through the request to the corresponding author (SMB).

## References

[1] Joint United Nations Programme on HIV/AIDS (UNAIDS) (2016). Global AIDS Update 2016: Global AIDS Update.

[2] World Health Organization (WHO) (2012). Guidance on Couples HIV Testing and Counselling—Including Antiretroviral Therapy for Treatment and Prevention in Serodiscordant Couples: Recommendations for a Public Health Approach.

[3] Carlsson-Lalloo E., Rusner M., Mellgren A., Berg M. (2016). Sexuality and reproduction in HIV-positive women: A meta-synthesis. AIDS Patient Care STDS..

[4] Cohen M.S., Chen Y.Q., McCauley M., Gamble T., Hosseinipour M.C., Kumarasamy N., Hakim J.G., Kumwenda J., Grinsztejn B., Pilotto J.H.S. (2016). Antiretroviral therapy for the prevention of HIV-1 transmission. N. Engl. J. Med..

[5] Rodger A.J., Cambiano V., Bruun T., Vernazza P., Collins S., Van Lunzen J., Corbelli G.M., Estrada V., Geretti A.M., Beloukas A. (2016). Sexual activity without condoms and risk of HIV transmission in serodifferent couples when the HIV-positive partner is using suppressive antiretroviral therapy. JAMA—J. Am. Med. Assoc..

[6] Gilling-Smith C., Nicopoullos J.D., Semprini A.E., Frodshan L.C. (2006). HIV and reproductive care—A review of current practice. BJOG.

[7] Narasimhan M., Celum C., Askew I., Kiarie J., van der Poel S. (2017). Supporting people living with HIV in serodiscordant partnerships to attempt a desired pregnancy by integrating sexual and reproductive health and HIV interventions. J. Int. AIDS Soc..

[8] Nóbrega A.A., Oliveira F.A., Galvão M.T., Mota R.S., Barbosa R.M., Dourado I., Kendall C., Kerr-Pontes L.R. (2007). Desire for a child among women living with HIV/AIDS in Northeast Brazil. AIDS Patient Care STDS.

[9] da Silveira R.A., Fonsechi-Carvasana G.A., Makuchb M.Y., Amarala E., Bahamondesa L. (2005). Factors associated with reproductive options in HIV-Infected women. Contraception.

[10] Yan X., Du J., Ji G.P. (2021). Prevalence and factors associated with fertility desire among people living with HIV: A systematic review and meta-analysis. PLoS ONE.

[11] Mekonnen B., Minyihun A. (2019). fertility desire and associated factors among HIV positive women attending art clinics in amhara region referral hospitals in Northwest Ethiopia, 2017. HIV/AIDS—Res. Palliat. Care.

[12] Niragire F., Ndikumana C., Nyirahabimana M.G., Uwizeye D. (2021). Prevalence and factors associated with fertility desire among HIV-positive women in rwanda in the context of improved life expectancy. Arch. Public Health.

[13] Cohn S.E., Haddad L.B., Sheth A.N., Hayford C., Chmiel J.S., Janulis P.F., Schmandt J. (2018). Parenting desires among individuals living with human immunodeficiency virus in the united states. Open Forum Infect. Dis..

[14] Hernando V., Alejos B., Álvarez D., Montero M., Pérez-Elías M.J., Blanco J.R., Masiá M., del Romero J., de los Santos I., Rio I. (2014). Reproductive desire in women with HIV Infection in Spain, associated factors and motivations: A mixed-method study. BMC Pregnancy Childbirth.

[15] Paiva V., Santos N., França-Junior I., Filipe E., Ayres J.R., Segurado A. (2007). Desire to have children: Gender and reproductive rights of men and women living with HIV: A challenge to health care in Brazil. AIDS Patient Care STDS.

[16] Rhodes C.M., Cu-Uvin S. (2016). Pregnancy desire, partner serodiscordance, and partner HIV disclosure among reproductive age HIV-infected women in an urban clinic. Infect. Dis. Obs. Gynecol..

[17] Mantell J.E., Exner T.M., Cooper D., Bai D., Leu C.-S., Hoffman S., Myer L., Moodley J., Kelvin E.A., Constant D. (2014). Pregnancy intent among a sample of recently diagnosed HIV-positive women and men practicing unprotected sex in Cape Town, South Africa. J. Acquir. Immune Defic. Syndr..

[18] Ramos De Souza M., Do Amaral W.N., Alves Guimarães R., Rezza G., Brunini S.M. (2017). reproductive desire among women living with HIV/AIDS in Central Brazil: Prevalence and associated factors. PLoS ONE.

[19] Eka P.O., Ujah I.O.A., Musa J., Swende T.Z., Achinge G., Maanongun M. (2016). Reproductive desires and intentions of HIV-positive women of reproductive age attending the adult HIV Clinic at the Jos University Teaching Hospital, Jos, Nigeria. Trop. J. Obstet. Gynaecol..

[20] Villela W.V., Barbosa R.M. (2015). Prevention of the heterosexual HIV infection among women: Is it possible to think about strategies without considering their reproductive demands?. Rev. Bras. Epidemiol..

[21] Farquhar C. (2010). Predicting pregnancy in HIV-1-discordant couples. AIDS Behav..

[22] World Health Organization (WHO) (2022). Women of Reproductive Age (15–49 Years) Population (Thousands).

[23] Jose H., Madi D., Chowta N., Ramapuram J., Bhaskaran U., Achappa B., Chandran V. (2016). Fertility desires and intentions among people living with HIV/AIDS (PLWHA) in Southern India. J. Clin. Diagn. Res..

[24] Ministério do Planejamento Orçamento e Gestão, Instituto Brasileiro de Geografia e Estatística, Diretoria de Pesquisas (2008). Coordenação de População e Indicadores Sociais Características étnico-raciais da população: Um estudo das categorias de classificação de cor ou raça. Brasília, DF: IBGE. http://biblioteca.ibge.gov.br/visualizacao/livros/liv49891.pdf.

[25] Cook R., Potter J.E., Miron-Shatz T., Chakhtoura N., Spence A., Byrne M.M. (2016). Fertility desires among women living with HIV. PLoS ONE.

[26] Carneiro P.B., Westmoreland D.A., Patel V.V., Grov C. (2021). Awareness and acceptability of undetectable = untransmittable among a u.s. national sample of HIV-negative sexual and gender minorities. AIDS Behav..

[27] Mohammed F., Assefa N. (2016). Determinants of desire for children among HIV-positive women in the Afar Region, Ethiopia: Case control study. PLoS ONE.

[28] Riccardi N., Rotulo G.A., Castagnola E. (2019). Definition of opportunistic infections in immunocompromised children on the basis of etiologies and clinical features: A summary for practical purposes. Curr. Pediatr. Rev..

[29] Demissie B.D., Tebeje B., Tesfaye T. (2014). Fertility desire and associated factors among people living with HIV attending antiretroviral therapy clinic in Ethiopia. BMC Pregnancy Childbirth.

[30] Gutin S.A., Namusoke F., Shade S.B., Mirembe F. (2014). Fertility desires and intentions among HIV-positive women during the post-natal period in Uganda. Afr. J. Reprod. Health.

[31] Ogilvie G.S., Palepu A., Remple V.P., Maan E., Heath K., MacDonald G., Christilaw J., Berkowitz J., Fisher W.A., Burdge D.R. (2007). Fertility intentions of women of reproductive age living with HIV in British Columbia, Canada. AIDS.

[32] Marcellin F., Protopopescua C., Abe C., Boyera S., Blanchea J., Ongolo-Zogoe P., Koulla-Shirof S., Moattia J.-P., Carrieria M.P., Spire B. (2010). Desire for a child among HIV-infected women receiving antiretroviral therapy in Cameroon: Results from the National Survey EVAL (ANRS 12–116). AIDS Care.

[33] Wekesa E., Coast E. (2014). Fertility Desires among men and women living with HIV/AIDS in Nairobi Slums: A mixed methods study. PLoS ONE.

[34] Kawale P., Mindry D., Stramotas S., Chilikoh P., Phoya A., Henry K., Elashoff D., Jansen P., Hoffman R. (2014). Factors associated with desire for children among HIV-infected women and men: A quantitative and qualitative analysis from malawi and implications for the delivery of safer conception counseling. AIDS Care.

[35] Beyeza-Kashesya J., Ekstrom A.M., Kaharuza F., Mirembe F., Neema S., Kulane A. (2010). My Partner Wants a Child: A Cross-Sectional Study of the Determinants of the desire for children among mutually disclosed sero-discordant couples receiving care in Uganda. BMC Public Health.

[36] Biseck T., Kumwenda S., Kalulu K., Chidziwisano K., Kalumbi L. (2015). Exploring fertility decisions among pregnant HIV-positive women on antiretroviral therapy at a health centre in Balaka, Malawi: A descriptive qualitative. Malawi Med. J..

[37] Kanniappan S., Jeyapaul M.J., Kalyanwala S. (2008). Desire for motherhood: Exploring HIV-positive women’s desires, intentions and decision-making in attaining motherhood. AIDS Care.

[38] Panozzo L., Battegay M., Friedl A., Vernazza P.L., Swiss Cohort Study (2003). High risk behaviour and fertility desires among heterosexual HIV-positive patients with a serodiscordant partner--two challenging issues. Swiss Med. Wkly.

[39] Mmbaga E.J., Leyna G.H., Ezekiel M.J., Kakoko D.C. (2013). Fertility desire and intention of people living with HIV/AIDS in Tanzania: A call for restructuring care and treatment services. BMC Public Health.

[40] da Silveira R.A., Amaral E., Makuch M.Y. (2011). Access of people living with HIV to infertility services: Perspective of brazilian healthcare professionals. AIDS Care.

